# Variations in the shape of foramen magnum at the base of human skulls among Indians in Rajasthan

**DOI:** 10.6026/97320630018488

**Published:** 2022-05-31

**Authors:** Raj Kumar, Hemant Ashish Harode, Rakesh Vora, Mayankkumar Javia

**Affiliations:** 1 Department of Anatomy, Shantabaa Medical College & General Hospital, Amreli, Gujarat 365601 India;; 2 Department of Anatomy, Zydus Medical College & Hospital, Dahod, Gujarat 389151 India

**Keywords:** Radiological study, CT Image, variations, shape, foramen magnum

## Abstract

Variations in the shape of foramen magnum can affect the normal anatomy of vital structures passing through it. Therefore, it is of interest to evaluate the various shapes of foramen magnum by using CT scans performed in patients of Indian population to establish clinical correlation. A total of 314 CT images of human skull base obtained from the Department of Radio-diagnosis, Geetanjali Medical College and Hospital, Udaipur, Rajasthan were used in the present study. All the patients’ CT scans were observed to determine the shape of foramen magnum. They were classified into one of the following shapes: Oval, round, tetragonal, egg shaped, hexagonal, pentagonal and irregular. The shapes of the foramen magnum in CT scans were oval in 39.09%, round in 22.61%, tetragonal in 12.10%, hexagonal in 10.51%, irregular in 7.96%, pentagonal in 5.41% and egg shaped in 1.59% CT images. Data shows that it is easy to operate at the base of skull in case of round, oval and hexagonal shape foramen magnum, as the working space is more in these shapes.

## Background:

In modern era of clinical medicine, CT scan has evolved as a powerful and widely used diagnostic imaging tool. [1] The newest and most advanced Computed Tomography (CT) and Magnetic Resonance Imaging (MRI) methods can evaluate anatomical variations of living human subjects. [2] Knowledge of the incidence of symmetric in variations of foramina of human skull is helpful in diagnostic evaluation of radiologic film. [3] Variations in shape of foramen magnum can affect the normal anatomy of vital structures passing through it. [4] Therefore, it is of interest to evaluate the various shapes of foramen magnum by using CT scans performed in patients of Indian population to establish clinical correlation.

## Materials and Methods:

Present study was done in the Department of Anatomy, Geetanjali Medical College and Hospital, Udaipur, Rajasthan after obtaining institutional ethical approval. Total 314 patients' CT images of human skull base obtained from the Department of Radio-diagnosis, Geetanjali Medical College and Hospital, Udaipur, Rajasthan were used in the present study. The CT images belong to the patients who had undergone CT scan evaluation for head and neck region for different clinical indications between the year of 2013 and 2018. All the patients' CT scans were visually assessed to determine the shape of FM and was classified into one of the following seven shapes - oval, round, tetragonal, egg shaped, hexagonal, pentagonal and irregular (Figure 1 - see PDF). The findings of the present study were statistically analyzed by using Microsoft Office Excel 2019.

## Results & Discussion:

The various shapes of the foramen magnum in CT images found in current study are oval in 39.09%, round in 22.61%, tetragonal in 12.10%, hexagonal in 10.51%, irregular in 7.96%, pentagonal in 5.41% and egg shaped in 1.59% cases. (Table 1, Figure 2 - see PDF) Evaluation of the different shape of skull base foramina by using computed tomography scans of patients has gained importance in clinical medicine. Most common shape of foramen magnum observed in the present study was oval, which was present in 39.09% cases. This value is lower as compared to the observations by Ganapathy et al. [5] (2014) (Table 2 - see PDF), who found oval shaped foramen magnum in 66% CT images. Furtado et al. [4] (2010) (Table 2 - see PDF) in 9.6 %, Sarthak et al. [6] (2016) (Table 2 - see PDF) in 18.2%, Edwards et al. [7] (2013) (Table 2 - see PDF) in 24.4% and Aghakhani et al. [8] (2016) (Table 2 - see PDF) in 35% CT images noticed oval shaped FM, which are lower than the findings of the present study. Round shape of foramen magnum was observed in 22.61% CT images. Edwards et al. [7] (2013) in 26% and Sarthak et al. [6] (2016) in 35.4% CT images observed round shape of foramen magnum, which are higher than the present value. The findings of following Authors are less than the present values: Furtado et al. [4] (2010) in 4.6%, Ganapathy et al. [5] (2014) in 9% and Aghakhani et al. [8] (2016) in 8% CT images. This may be due to variation in the categorization of oval and round shapes because percentages of oval shape foramen magnum has decreased and reciprocal increase in the percentage of round shape foramen magnum. In the present study, the foramen magnum was tetragonal shape in 12.10% CT images. Furtado et al. [4] (2010) observed tetragonal shape of foramen magnum in 19.04% CT images in his study, which is greater than the present value. Ganapathy et al. [5] (2014) in 9%, Edwards et al. [7] (2013) in 6%, Aghakhani et al. [8] (2016) in 4.4% Sarthak et al. [6] (2016) in 8% CT images observed tetragonal shape of foramen magnum, which are lower than the current study. In the present study the foramen magnum was hexagonal shape in 10.51% CT images. Furtado et al. [4] (2010) in 19.04 %, Edwards K et al. [7] (2013) in 16.4% and Aghakhani et al. [8] (2016) in 23% CT images in their study, which is greater than the present value whereas Ganapathy et al. [5] (2014) observed hexagonal shape of foramen magnum in 10 % CT images in their study, which is less than present value. In the present study the foramen magnum was of irregular shape in 7.96 % CT images. The findings of following authors were higher than the present value: Ganapathy et al. [5] (2014) in 16%, Sarthak et al. [6] (2016) in 25.8%, Edwards et al. [7] (2013) in 15% and Aghakhani et al. [8] (2016) in 12.4% CT images. In the present study, the foramen magnum was of pentagonal shape in 5.41% CT images. Furtado et al. [4] (2010) in 9.6 %, Sarthak et al. [6] (2016) in 11.8%, Edwards et al. [7] (2013) in 8.4 % and Aghakhani et al. [8] (2016) in 13% CT images observed pentagonal shape of foramen magnum. These values are greater than the present value. In the present study, the foramen magnum was egg shaped in 1.59% cases. Furtado et al. [4] (2010) in 9.5 % and Edwards et al. [7] (2013) in 9.5% CT images observed egg shape of foramen magnum in their study, which is higher than the present value. The difference in percentages of various shapes of foramen magnum could be due to study done on different population or inter-observer variation or genetic variation. Anatomical values obtained by different authors are nearly the same, which is not correct with regards to the values of the radiologic study [9-14].

## Conclusion:

Data shows that the oval shape is the most common, round is the second most common and egg shape is the least most common shape of foramen magnum in CT images of Indian population. Data also shows that it is easy to operate at the base of skull in case of round, oval and hexagonal shape foramen magnum, as the working space is more in these shapes.
